# Quantification of parasite clearance in *Plasmodium knowlesi* infections

**DOI:** 10.1186/s12936-023-04483-9

**Published:** 2023-02-14

**Authors:** Jeyamalar T. Thurai Rathnam, Matthew J. Grigg, Saber Dini, Timothy William, Sitti Saimah Sakam, Daniel J. Cooper, Giri S. Rajahram, Bridget E. Barber, Nicholas M. Anstey, Ali Haghiri, Megha Rajasekhar, Julie A. Simpson

**Affiliations:** 1 Centre for Epidemiology and Biostatistics, Melbourne School of Population and Global Health, The University of Melbourne, Melbourne, Australia; 2 Menzies School of Health Research and Charles Darwin University, Darwin, NT Australia; 3 Infectious Diseases Society Sabah-Menzies School of Health Research Clinical Research Unit, Kota Kinabalu, Sabah, Malaysia; 4 Clinical Research Centre, Queen Elizabeth II Hospital, Ministry of Health, Kota Kinabalu, Sabah, Malaysia; 5 Department of Medicine, School of Clinical Medicine, University of Cambridge, Cambridge, UK; 6 QIMR Berghofer Medical Research Institute, Brisbane, Australia

**Keywords:** Malaria, Parasite clearance rate, *Plasmodium knowlesi*, Bayesian hierarchical modelling

## Abstract

**Background:**

The incidence of zoonotic *Plasmodium knowlesi* infections in humans is rising in Southeast Asia, leading to clinical studies to monitor the efficacy of anti-malarial treatments for knowlesi malaria. One of the key outcomes of anti-malarial drug efficacy is parasite clearance. For *Plasmodium falciparum*, parasite clearance is typically estimated using a two-stage method, that involves estimating parasite clearance for individual patients followed by pooling of individual estimates to derive population estimates. An alternative approach is Bayesian hierarchical modelling which simultaneously analyses all parasite-time patient profiles to determine parasite clearance. This study compared these methods for estimating parasite clearance in *P. knowlesi* treatment efficacy studies, with typically fewer parasite measurements per patient due to high susceptibility to anti-malarials.

**Methods:**

Using parasite clearance data from 714 patients with knowlesi malaria and enrolled in three trials, the Worldwide Antimalarial Resistance Network (WWARN) Parasite Clearance Estimator (PCE) standard two-stage approach and Bayesian hierarchical modelling were compared. Both methods estimate the parasite clearance rate from a model that incorporates a lag phase, slope, and tail phase for the parasitaemia profiles.

**Results:**

The standard two-stage approach successfully estimated the parasite clearance rate for 678 patients, with 36 (5%) patients excluded due to an insufficient number of available parasitaemia measurements. The Bayesian hierarchical estimation method was applied to the parasitaemia data of all 714 patients. Overall, the Bayesian method estimated a faster population mean parasite clearance (0.36/h, 95% credible interval [0.18, 0.65]) compared to the standard two-stage method (0.26/h, 95% confidence interval [0.11, 0.46]), with better model fits (compared visually). Artemisinin-based combination therapy (ACT) is more effective in treating *P. knowlesi* than chloroquine, as confirmed by both methods, with a mean estimated parasite clearance half-life of 2.5 and 3.6 h, respectively using the standard two-stage method, and 1.8 and 2.9 h using the Bayesian method.

**Conclusion:**

For clinical studies of *P. knowlesi* with frequent parasite measurements, the standard two-stage approach (WWARN’s PCE) is recommended as this method is straightforward to implement. For studies with fewer parasite measurements per patient, the Bayesian approach should be considered. Regardless of method used, ACT is more efficacious than chloroquine, confirming the findings of the original trials.

**Supplementary Information:**

The online version contains supplementary material available at 10.1186/s12936-023-04483-9.

## Background

In recent years, there has been a rise in reported *Plasmodium knowlesi* malaria cases in Southeast Asia with 435 cases reported in Philippines, Thailand and Indonesia [1]. In Malaysia, where *P. knowlesi* is the predominant cause of human malaria, there were 3575 known cases in 2021 with 13 deaths reported [1, 2]. *Plasmodium knowlesi* is a zoonotic malaria with natural macaque hosts [3–7]. Unlike *Plasmodium* species that are transmitted from human-to-human, such as *Plasmodium falciparum* and *Plasmodium vivax*, transmission of *P. knowlesi* parasites occurs from macaques to mosquitoes to humans and there is no clear evidence of natural human-mosquito-human transmission [8, 9].

*Plasmodium knowlesi* has a 24-h asexual life cycle within the red blood cell of an infected human, which is shorter than that of human-only *Plasmodium* species, which typically range between 48- and 72-h cycles [10]. Human red blood cell invasion by *P. knowlesi* is mediated by binding to Duffy antigen/chemokine receptors present on young reticulocytes [11]. However, alternative pathways involving normocyte binding proteins along with the short life cycle may play a role in the rapid development of high parasite levels seen in a minority of *P. knowlesi* infections [12, 13]. Age-dependent effects on parasite biomass, endothelial activation and inflammation contribute to the higher risk of severe disease seen in *P. knowlesi* infections [14] at lower parasitaemia levels compared to *P. falciparum* (median 42,000 parasites/μL versus > 100,000 parasites/μL of blood) [15, 16]. The high case fatality rate (2.45/1000 notifications in Sabah, Malaysia) [17] highlights the importance of prompt diagnosis and efficacious anti-malarial treatment for *P. knowlesi* infections.

Although the World Health Organization (WHO) suggests either artemisinin-based combination therapy (ACT) or chloroquine for the treatment of uncomplicated *P. knowlesi* infections [18], ACT is recommended [19] because of greater efficacy and the potential for microscopic misdiagnosis of *P. falciparum* or *P. vivax* as *P. knowlesi*, as the former two species are chloroquine-resistant in knowlesi-endemic areas [20, 21]. For severe knowlesi malaria, the recommended treatment is parenteral artesunate, followed by a full course of oral ACT [18]. Clinical trials have shown that ACT, whether using artemether–lumefantrine or artesunate–mefloquine, is more efficacious in treating uncomplicated *P. knowlesi* infections compared to chloroquine, including a lower risk of anaemia [22, 23]. While there is no evidence of anti-malarial drug resistance for *P. knowlesi* [24, 25], continual monitoring of the efficacy of anti-malarials, including initial clearance of parasites, is crucial to ensure that the best treatment regimen is used.

For the quantification of parasite clearance following treatment of *P. falciparum* infections, the Worldwide Antimalarial Resistance Network’s (WWARN) Parasite Clearance Estimator (PCE) [26, 27] is widely used. This method involves two stages, the first of which requires estimating parasite clearance for each patient parasite profile separately, therefore requiring frequent parasite measurements above the level of detection in the parasite clearance phase. For *P. knowlesi* infections, patients may initially present with a high parasite load which rapidly decreases and reaches below the detection limit sooner than for human only species like *P. falciparum*, resulting in fewer data points per patient for parasite clearance estimation. This poses a challenge as many patients may be excluded from the analysis, potentially leading to selection bias when calculating the population mean parasite clearance rate in the second stage of the analysis.

Alternatively, all patient parasite measurement profiles could be analysed simultaneously using a Bayesian hierarchical framework [28, 29]. This method is flexible in that it allows for unbalanced or sparse sampling designs, which means all patient profiles will contribute to estimating the population mean parasite clearance rate, thereby, reducing bias [29, 30]. However, the implementation is computationally complex and there may be convergence issues if there is too much variation between patients in parasite clearance profiles.

In previous studies, these two methods have been compared and used for the estimation of parasite clearance rate by considering lag, decay and tail phases in patient parasite clearance profiles to enable the detection of artemisinin resistance in patients with *P. falciparum* [27–29, 31–34]. Despite the Bayesian hierarchical approach showing improved statistical properties (bias, precision and coverage) in a simulation study, the standard two-stage method is widely used in practice for clinical efficacy studies of falciparum malaria because it is simpler and computationally faster [29, 30]. Also, the availability of datasets with rich sampling (> 5 parasitaemia measurements above the detection limit) for patients with *P. falciparum* (due to anti-malarial drug resistance and a longer 48-h erythrocytic life-cycle) ensures that the potential selection bias associated with the two-stage approach is minimal. On the contrary, *P. knowlesi* is still highly susceptible to current anti-malarials, resulting in rapidly decreasing parasitaemia post-drug administration, potentially limiting the richness of available data. This study aimed to compare the two-stage WWARN PCE with the Bayesian hierarchical method for estimating parasite clearance rate in anti-malarial efficacy studies of *P. knowlesi*.

## Methods

### Data source

Parasitaemia data from 714 patients enrolled in three clinical trials, ACT KNOW [22, 37], CAN KNOW [23], and PACKNOW [35, 36] were analysed in this study. The patients enrolled in the ACT KNOW (comparing anti-malarial treatments, artesunate–mefloquine and chloroquine) and CAN KNOW (comparing treatments, artemether–lumefantrine and chloroquine) trials were predominantly adults, although also included children (aged ≥ 3 years) with uncomplicated *P. knowlesi* malaria. The PACKNOW (determining the effects of regularly dosed paracetamol on renal function in *P. knowlesi* malaria) trial included adults and children aged ≥ 12 years with *P. knowlesi* malaria of any severity. A total of 16 patients enrolled in the PACKNOW trial were initially given one dose of artemether–lumefantrine, before the diagnosis of severe malaria with subsequent administration of intravenous artesunate.

The malaria diagnosis for patients was confirmed using a validated polymerase chain reaction (PCR). Patients who were pregnant, lactating, had non-*P. knowlesi* malaria infections or serious underlying health conditions were excluded. The parasite counts were measured using microscopy at time 0 and every 6 h following anti-malarial drug administration until the parasitaemia fell below the microscopic detection limit (where possible). The thick and thin blood films were obtained from patients via the finger prick method [37]. The microscopic slides were read by an independent microscopist to determine the parasite count, without knowing the antimalarial treatments used. The slides were checked by an ‘expert microscopist’ and the counts obtained were used [37]. Any disagreements in the counts were examined by a third microscopist and the results were obtained by taking an average of the two closest parasite counts [37]. The clinical trials were conducted between 2012 and 2018 in Sabah, Malaysia (see Table 1 for further details).

**Table 1:** Clinical trials data analysed in this study

Trial details	Data used in this study (714 patients)		
Trial name (reference)	ACT KNOW [22]	CAN KNOW [23]	PACKNOW [35, 36]
Trial duration	October 2012 – December 2014	January 2014 – March 2015	October 2016 – February 2018
Number of patients	226	123	365
Location	Sabah, Malaysia Kota Marudu District Hospital Kudat District Hospital Pitas District Hospital	Sabah, Malaysia Kota Marudu District Hospital Kudat District Hospital Pitas District Hospital	Sabah, Malaysia Keningau District Hospital Queen Elizabeth Hospital Kota Marudu District Hospital Ranau District Hospital
Study population	Children (age $\ge$ 3 years and weight > 10 kg) and adults with uncomplicated *P. knowlesi* malaria	Children (age $\ge$ 4 years and weight > 10 kg) and adults with uncomplicated *P. knowlesi* malaria	Children (age $\ge$ 12 years) and adults with uncomplicated and severe *P. knowlesi* malaria
Anti-malarial treatment	Artesunate–mefloquine Chloroquine	Artemether–lumefantrine Chloroquine	Artemether–lumefantrine Intravenous artesunate followed by artemether–lumefantrine Artemether–lumefantrine followed by intravenous artesunate
Parasitaemia sampling times	Initial sample (time = 0) and every 6 h post anti-malarial drug administration until the parasitaemia fell below the microscopic detection limit for two consecutive samples, where possible		

### Statistical approaches

The two-stage approach adopted by the WWARN PCE was compared with the Bayesian hierarchical framework. Both methods are described in more detail below.

### Method 1: Two-stage WWARN PCE

**Fig. 1:**
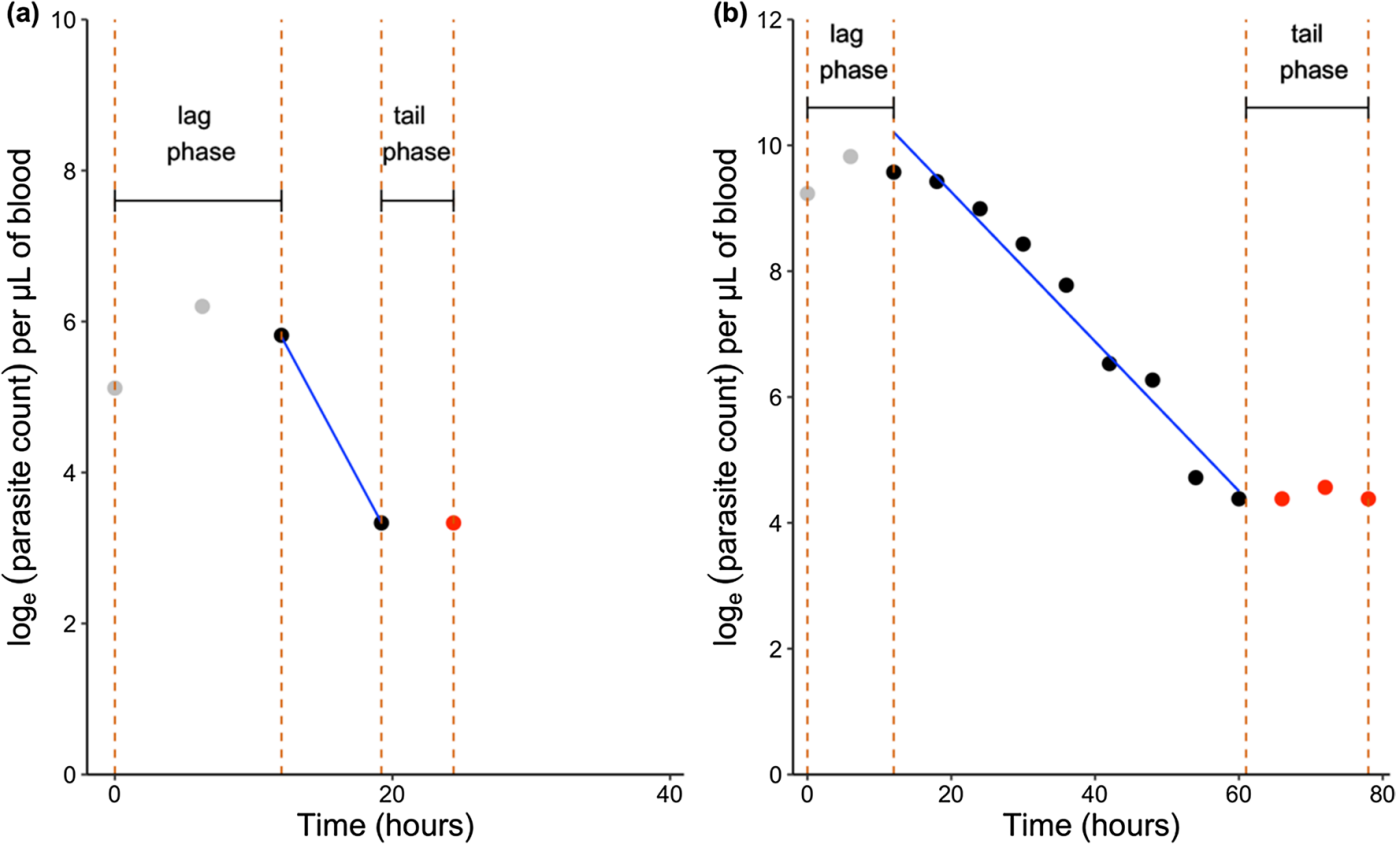
An example of a patient profile for *P. knowlesi* (**a**) and *P. falciparum* (**b**) [28, 33]

This method is frequently used to estimate parasite clearance to determine anti-malarial drug resistance for *P. falciparum* malaria [31–34, 38]. The first stage involves fitting a log-linear, log-quadratic, or log-cubic regression model depending on the parasitaemia profile, to parasitaemia data of each individual patient separately [27]. This method works best when 6-hourly measurements of parasitaemia are taken [27, 39], and there are at least three data points in the parasite clearance phase. Parasitaemia measurements selected for the lag or tail phases of the parasitaemia profile are excluded (Fig. 1) to prevent inaccurate estimations of parasite clearance half-lives [27].

The occurrence and duration of lag phase depends on different factors, including the rate of drug absorption and the distribution of parasites across the erythrocytic life cycle at the time of treatment [29]. The tail phase generally occurs when the parasite density tapers out above the detection limit before falling below the detection limit. The half-life of parasite clearance (hours) is calculated from the negative slope of the predicted regression line for log-parasitaemia over time [27]. The first stage can be performed using the WWARN PCE R code [27] or the WWARN PCE online tool [26].

The second stage involves estimation of the median and/or mean parasite clearance rate for the population using the individual parasite clearance rate estimates obtained in the first stage. The effects of anti-malarial treatment administered and patient/parasite factors such as age, sex, and biological markers on the parasite clearance rate are determined using second-level regression where the outcome variable is the individual estimates of parasite clearance rate derived in the first stage. Further details regarding this method are available in Flegg et al. [27].

### Method 2: Bayesian hierarchical modelling

The Bayesian hierarchical modelling approach accommodates individual- and population-level parameters in the model. Patient and parasite features can also be included as covariates to investigate the effect of these factors on parasite clearance. This method incorporates the two-stages described for Method 1 concurrently and uses a changepoint model (Eq. 1) within the hierarchical model to determine the occurrence of lag and tail phases.

**Fig. 2:**
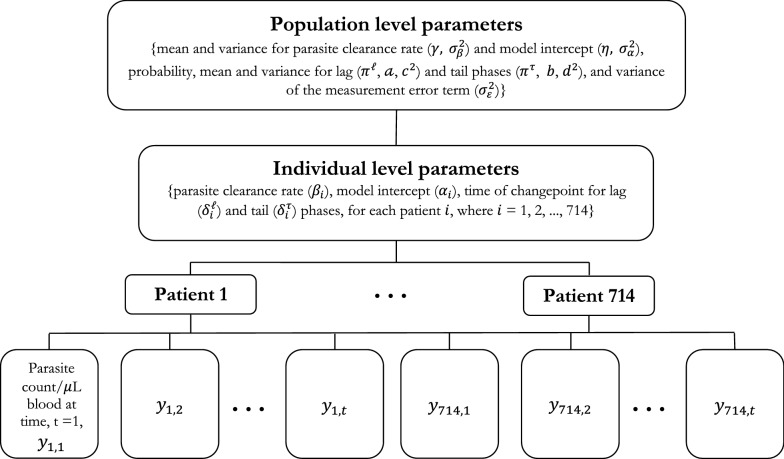
The hierarchical structure used in the Bayesian hierarchical model (Method 2)

Figure 2 depicts a typical multilevel Bayesian structure. The distribution for the parasite density,${y}_{ij}$, for each patient i = 1, …,714 at time points, j = 1, …, t is determined by the individual level parameters, i.e. the parasite clearance rate, model intercept and time of changepoint for the lag and tail phases, which is unique for each patient. Similarly, the population level parameters describe the distribution for the individual level parameters. These parameters also have a set of prior distributions.

The model (Eq. 1) used for parasite density (${y}_{ij}$) is:

1 $$\text{log }\left( {{y}_{ij}} \right) = {{\alpha }_{i}}-{{\beta }_{i}} (\delta _{i}^{\ell }\mathbbm{1}\{{{t}_{ij}}<\delta _{i}^{\ell }\}+{{t}_{ij}}\mathbbm{1}\left\{ \delta _{i}^{\ell }\le {{t}_{ij}}\le \delta _{i}^{\tau } \right\}+\delta _{i}^{\tau }\mathbbm{1}\{{{t}_{ij}}>\delta _{i}^{\tau }\})+{{\varepsilon }_{ij}},$$

The indicator variables $\mathbbm{1}\{{t}_{ij}<{\delta }_{i}^{\mathcal{l}}$} and $\mathbbm{1}\{{t}_{ij} > {\delta }_{i}^{\tau }$} are used to determine the existence of lag and/or tail phases, i.e. ${\delta }_{i}^{\mathcal{l}}$, the changepoint time between the lag and clearance phase, and ${\delta }_{i}^{\tau }$, the changepoint time between the clearance and decay phase. The parameter ${\alpha }_{i}$ denotes the model intercept, and ${\beta }_{i}$ is the parasite clearance rate for patients, while ${\varepsilon }_{ij}$ is the error term at the individual level [29]. For more detailed information regarding this model, please refer to Fogarty et al.[29] and Sharifi-Malvajerdi et al. [28].

The model was fit to the data using Markov chain Monte Carlo (MCMC) sampling of the posterior distributions with the Metropolis–Hastings-within-Gibbs sampler [28, 29]. Bayesian hierarchical modelling provides a more accurate estimation of the between- and within-patient variability, since parasitaemia data from all patients are analysed simultaneously. The patient profiles with more parasitaemia measurements contribute information to profiles with fewer sample measurements for the parasite clearance rate estimation, instead of omitting those profiles from the analysis.

### Data analysis

Data cleaning and all statistical analyses were performed using the open-source R software, version 4.1.0 (via RStudio software version 1.4.1717). Data from the three clinical studies were pooled for the purposes of this analysis, giving a total of 714 patients.

Method 1: The online tool for parasite clearance estimation by Flegg et al. [27] was used to obtain the parasite clearance rate constants and plots for patient profiles. The lower detection limit was set at 14 parasites/μL of blood and three parasitaemia measurements above the detection limit after the lag phase were required within the first 24 h [27]. The method also stipulates that the last parasitaemia measurement above the detection limit should not be more than 10,000 parasites/μL of blood, which is the upper limit for low-density parasitaemia. Following the analysis, visual checks were performed for the observed and predicted parasitaemia versus time plots obtained for each patient. The summary statistics of demographic and clinical characteristics for patients with successful and unsuccessful estimation of parasite clearance rate was presented to determine if there was any selection bias. For those patients with successful estimation of parasite clearance rate in stage 1, the mean parasite clearance rate constant (95% confidence interval (CI)) overall and by treatment arm was calculated.

Method 2: The R code from the ‘bhrcr’ package by Sharifi-Malvajerdi et al. [28] was used to obtain the population posterior median and corresponding 95% credible interval (CrI) for the parasite clearance rate. The normal distribution was chosen as the prior distribution for the log parasite clearance rate and model intercept, while Jeffrey’s prior was used for the error term [29]. The probability of existence of lag and tail phases followed a Beta(1,1) prior distribution [29]. 15,000 posterior samples were generated. The first 5000 samples were discarded as burn-in and only one of every 50 samples were kept, resulting in 200 samples per parameter for calculation of posterior summaries. The posterior summaries calculated were the median of the 200 samples for each parameter (posterior median) and 95% CrI, which is calculated from the 2.5th and 97.5th percentiles of the 200 samples. As for Method 1, a detection limit of 14 parasites/μL of blood was used. Trace plots were examined to assess the convergence of the 10,000 parameter draws to a common distribution for each parameter. Visual checks of the predicted and observed parasitaemia profiles per patient are also presented. To estimate the effect of treatment on parasite clearance, the anti-malarial treatment administered was included as a covariate on the parameter parasite clearance rate.

## Results

The 714 patients had a mean age of 37 years (9.2% aged ≤ 15 years), 579 (81%) were male, and the median parasitaemia prior to treatment was 1898 parasites/μL of blood (inter-quartile range (IQR): 463 to 6276 parasites/µL).

**Fig. 3:**
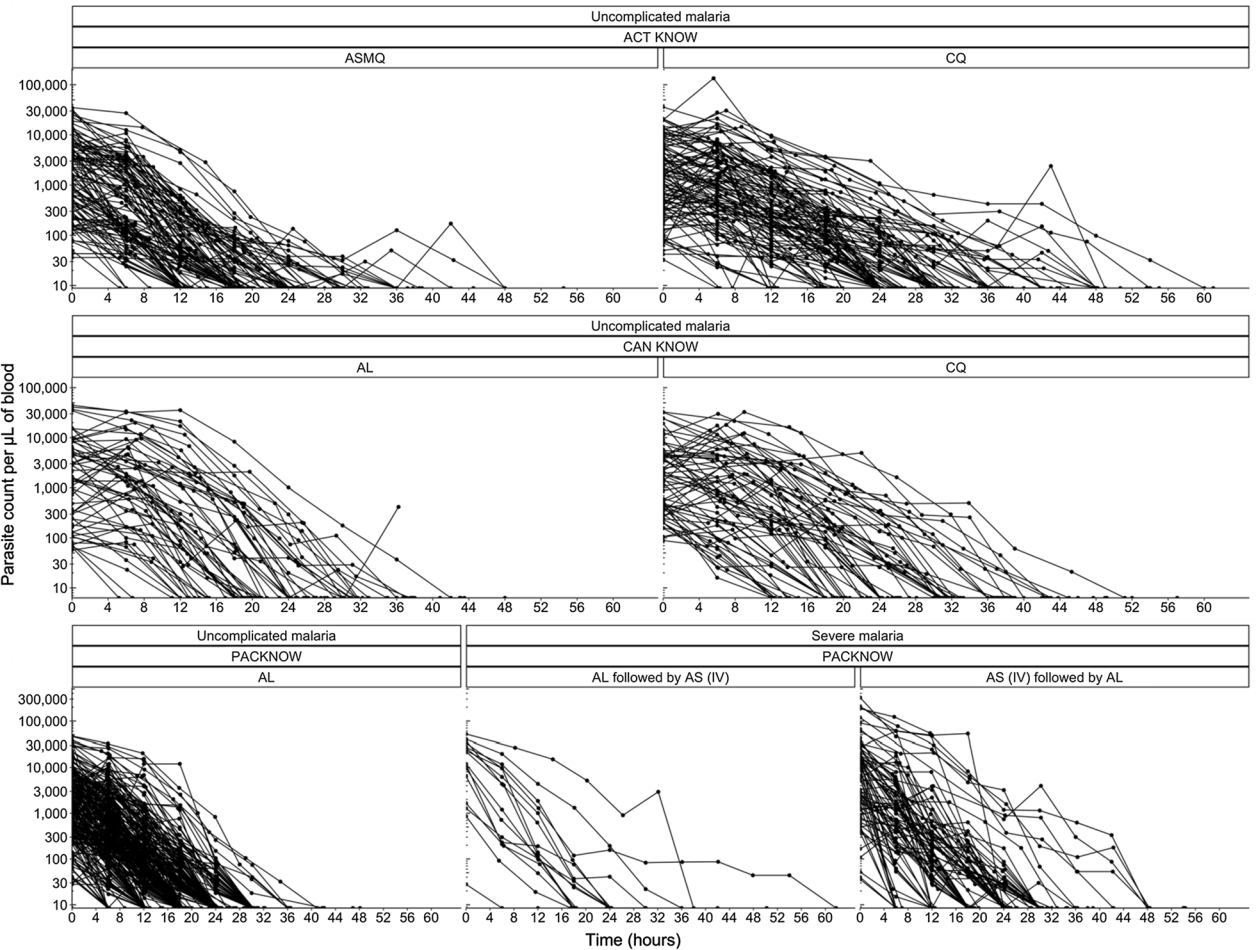
Patient parasitaemia profiles split by ACT KNOW, CAN KNOW and PACKNOW clinical trials, treatment arm and disease severity (AL - artemether-lumefantrine, ASMQ - artesunate-mefloquine, AS - artesunate, CQ - chloroquine, IV - intravenous)

Figure 3 shows the measured parasite count profiles following treatment for all patients, grouped by disease severity, clinical trial and anti-malarial drug treatment administered. About 20% of the patient profiles had an initial increase in parasitaemia before decreasing below the detection limit. The parasite counts for most patients fell below the detection limit within 60 h post anti-malarial drug treatment.

For the 714 individual patient parasitaemia profiles analysed using the two-stage WWARN PCE (Method 1), the parasite clearance rate constant was estimated for 678 (95%) patients’ individual parasitaemia profiles. The main reason for the exclusion of 36 patient parasitaemia profiles was the low number of parasite measurements available, with a median of 3 measurements (including measurements below the detection limit of 14/μL) per patient compared with 6 measurements per patient for the other 678 patients. Most of the excluded profiles only had one positive parasitaemia reading before parasitaemia fell below the microscopic detection limit, meaning that no slope for the parasite clearance phase was attainable. Table 2 provides patient characteristics for profiles where parasite clearance rate was successfully estimated and for those excluded.

**Table 2:** Characteristics for patient profiles analysed in stage 1 of the WWARN PCE^a^

Characteristics	All patients (n = 714 patients)	Successful analysis (n = 678 patients)	Unsuccessful analysis (n = 36 patients)
Number of parasite measurements			
Median, IQR	5, 5–6	6, 5–7	3, 3–3
Range	3–14	3–14	3–5
< 3	0	0	0
3	37 (5.2%)	2 (0.3%)	35 (97.2%)
4	107 (15%)	107 (15.8%)	0
5	217 (30.4%)	216 (31.9%)	1 (2.8%)
6	182 (25.5%)	182 (26.8%)	0
7	110 (15.4%)	110 (16.2%)	0
≥ 8	61 (8.5%)	61 (9%)	0
Sex			
Female	135 (18.9%)	128 (18.9%)	7 (19.4%)
Male	579 (81.1%)	550 (81.1%)	29 (80.6%)
Age (years)			
Median, IQR	35, 24–49	34, 23–48	37, 23–44.5
Range	3–96	3–96	14–68
Weight (kg)			
Median, IQR	58, 50–67	58, 50.4–67	59, 51.5–68
Range	11–118	11–118	35–80.5
Previous malaria			
No	511 (71.6%)	489 (72.1%)	22 (61.1%)
Yes	203 (28.4%)	189 (27.9%)	14 (38.9%)
Days of preceding fever			
Median, IQR	4, 3–7	4, 3–7	4, 3–7
Range	0–44^b^	0–44^c^	2–14^d^
Initial parasitaemia (/μL of blood)			
Median, IQR	1898, 463–6276	2056, 533–6398	127, 56–555
Range	28–325,294	28–325,294	28–16,748
Treatment			
Uncomplicated malaria			
Artemether–lumefantrine	320 (44.8%)	301 (44.3%)	19 (52.8%)
Artesunate–mefloquine	115 (16.1%)	111 (16.4%)	4 (11%)
Chloroquine	176 (24.7%)	174 (25.7%)	2 (5.6%)
Severe malaria			
Artemether–lumefantrine followed by artesunate (intravenous)	16 (2.2%)	15 (2.2%)	1 (2.8%)
Artesunate (intravenous) followed by artemether–lumefantrine	87 (12.2%)	77 (11.4%)	10 (27.8%)

^a^Worldwide Antimalarial Resistance Network’s Parasite Clearance Estimator

IQR: inter-quartile range; kg: kilograms; μL: microlitre

^b^18 patients did not have data for days of preceding fever

^c^2 patients did not have data for days of preceding fever

^d^16 patients did not have data for days of preceding fever

The distribution of age, sex and weight was similar for those patients with successful parasite clearance rate estimation and for those excluded. Only 28.4% of the patients included in the analysis had a self-reported previous malaria infection while there was a higher prevalence (39%) of patients with previous malaria infections in those excluded. Most patients (52.8%) excluded from the analysis were treated with artemether–lumefantrine (AL), compared with 44.3% for those with a successful parasite clearance estimation. Patients with successful estimation of parasite clearance rate had a higher median initial parasitaemia of 2056 parasites/μL compared to the excluded patients, who had a much lower median count of 127 parasites/μL.

**Fig. 4:**
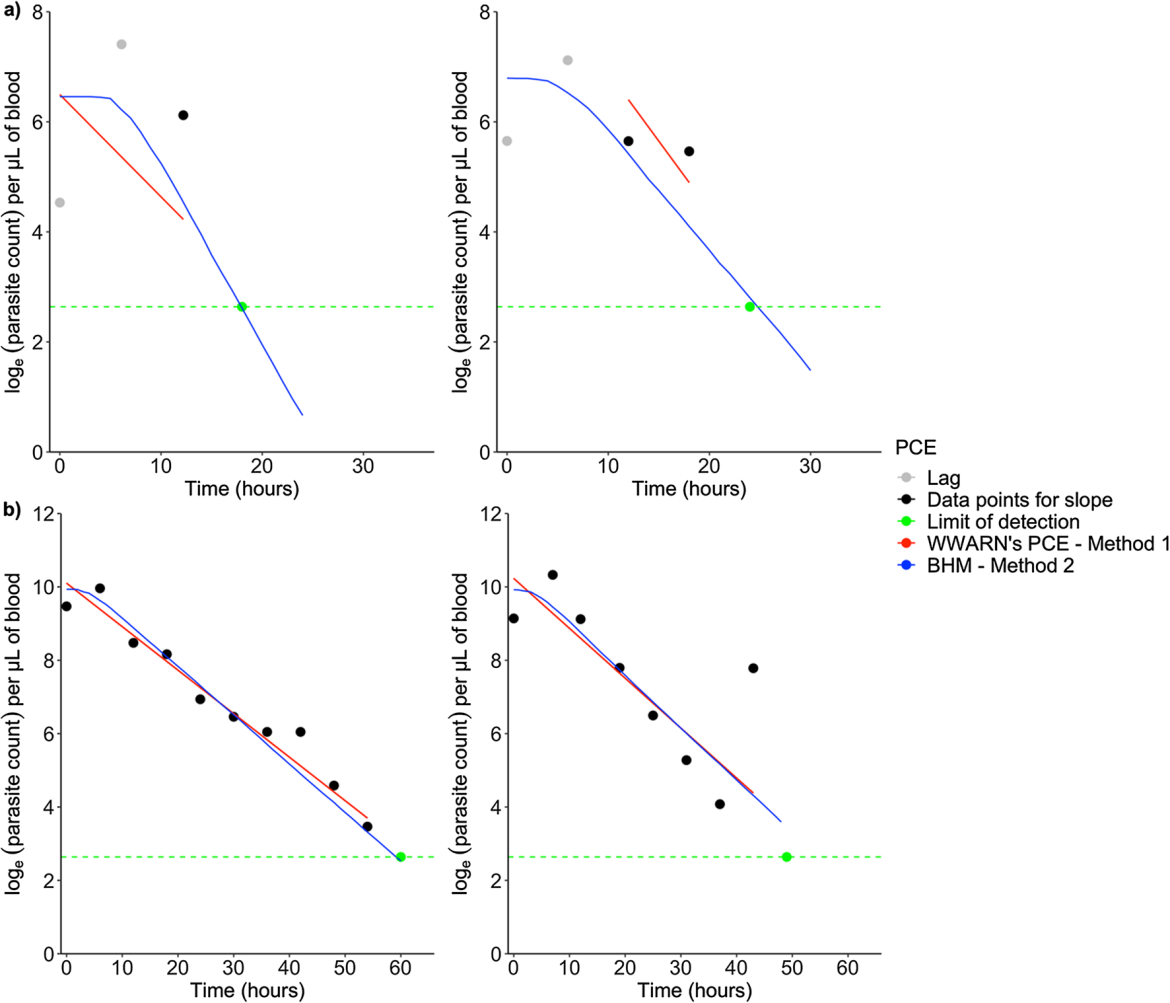
Plots of profiles analysed using WorldWide Antimalarial Resistance Network Parasite Clearance Estimator (WWARN PCE) and Bayesian hierarchical models (BHM). The top (**a**) and bottom (**b**) panels present two distinct patient profiles with ≤ 5 parasitaemia measurements per patient and > 7 parasitaemia measurements per patient, respectively

For Method 2, the data from all 714 patients were included in the analysis, pooled across the population regardless of treatment type. The diagnostic plots for Method 2 indicate that stationarity of the Markov chain for each parameter was obtained (Additional file 1: Figs. S1 and S2). Based on visual checks, both methods had good and poor fits to the individual parasitaemia profiles, which varied depending on the number of parasitaemia measurements above the limit of detection. The plots for patient profiles that were successfully analysed in stage 1 of Method 1 generally had poorer model fits to the observed parasitaemia data compared to the plots obtained for Method 2 (Fig. 4). There could be several reasons for this, including that the WWARN PCE method has been optimized for *P. falciparum* data, which generally tend to have a greater number of parasite measurements during the clearance phase than were available in the dataset. In the current pooled *P. knowlesi* dataset, only 75% of patients had between 4 to 6 parasite measurements. Another contributing factor could be the use of a changepoint model in Method 2, which allows for a more accurate lag, decay, and tail phase determination. A small number of model fits for Method 2 were also poor, possibly due to large between-patient variation. Figures 4a and 4b show examples of individual parasitaemia profiles and model fits from Methods 1 and 2.

In this study, lag phases were detected in 16% of patient profiles, while only 3% had tail phases. The mean population parasite clearance rate constant was 0.26/h (95% CI 0.11, 0.46) for Method 1 and 0.36/h (95% CrI 0.18, 0.65) for Method 2, corresponding to mean parasite clearance half-lives of 2.6 and 1.9 h, respectively. Further details are provided in Additional file 1: Table S1.

**Fig. 5:**
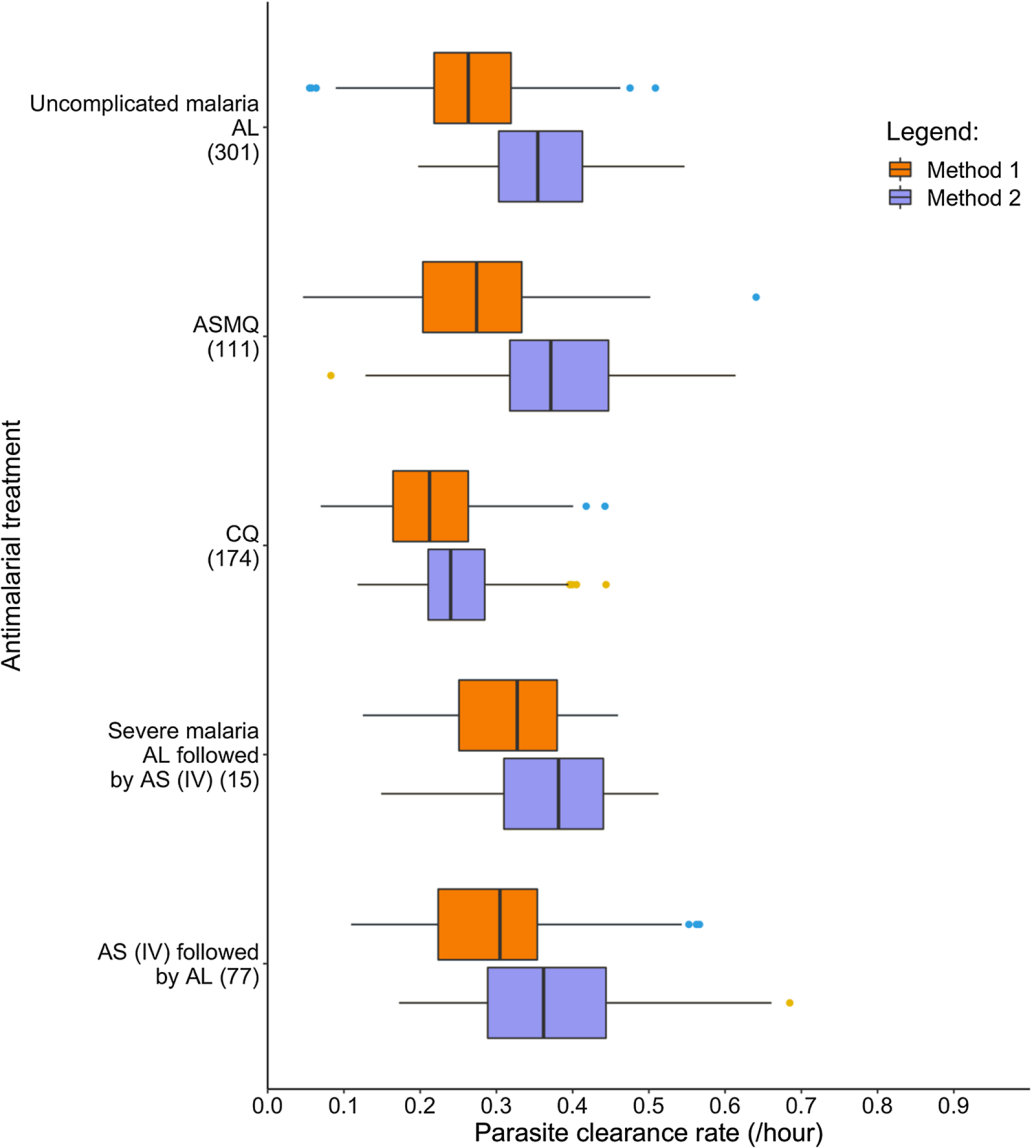
Box and whiskers plot for parasite clearance rate estimates, grouped by disease severity and treatment type (AL - artemether-lumefantrine, ASMQ - artesunate-mefloquine, AS - artesunate, CQ - chloroquine, IV - intravenous)

A further analysis was performed where the data were grouped by the five anti-malarial treatments. To enable a fair comparison using boxplots (Fig. 5), the patient groups were limited to only those that were analysed by both methods successfully (n = 678). Patients with uncomplicated *P. knowlesi* malaria were treated with artemether–lumefantrine (n = 301), artesunate–mefloquine (n = 111) and chloroquine (n = 174), and for severe *P. knowlesi* malaria patients, the treatments were artemether–lumefantrine (single dose) followed by intravenous artesunate (n = 15), and intravenous artesunate followed by artemether–lumefantrine (n = 77). The distribution of the individual-level estimated parasite clearance rate constants are presented by estimation method, disease severity, and treatment administered (Fig. 5).

The estimated parasite clearance rates using Method 2 are higher than Method 1 for all patient treatment groups (Fig. 5). Distribution of the parasite clearance rates for patients with uncomplicated malaria indicated that patients treated with artesunate–mefloquine and artemether–lumefantrine had faster parasite clearance rates compared to those treated with chloroquine, regardless of analysis method. Patients with severe malaria treated with intravenous artesunate followed by artemether–lumefantrine had similar distributions of parasite clearance rate to those receiving artemether–lumefantrine followed by intravenous artesunate, however, only 15 patients were administered the latter treatment sequence.

The mean, median and interquartile ranges for the parasite clearance half-lives (and parasite clearance rates) grouped by method used, disease severity and treatment type are given in Table 3 (Additional file 1: Table S2). The tables include the respective number of patients that were successfully analysed using each method to present results of the analysis in its entirety. This means that patients excluded following analysis using Method 1 were not removed from the results for Method 2. Consistent with the estimated parasite clearance rates presented in Fig. 5 (restricted to 678 patients analysed by both methods), the longest parasite clearance half-life for uncomplicated malaria estimated using Method 2 (analysis of all 714 patient data) was for patients receiving chloroquine (median of 2.9 h), compared with 1.9 h for patients receiving artesunate–mefloquine and artemether–lumefantrine. For patients with severe malaria, using Method 2, those treated with artemether–lumefantrine followed by intravenous artesunate had a similar distribution of parasite clearance half-life (median of 2.0 h and a 95% CrI [1.6, 2.4] hours) to those treated with intravenous artesunate followed by artemether–lumefantrine (median 1.8 h, 95% CrI [1.6, 2.0] hours).

**Table 3:** Estimated parasite clearance half-lives (hours) by anti-malarial treatment administered using WWARN PCE^a^ and Bayesian hierarchical modelling

Treatment	WWARN PCE^a^	Bayesian hierarchical modelling
*Uncomplicated* malaria		
Artemether–lumefantrine (n)	301	320
Mean (hours)	2.845	1.940
Median (hours)	2.634	1.940
95% CI & CrI (hours)	[1.568, 5.254]	[1.808, 2.116]
Artesunate–mefloquine (n)	111	115
Mean (hours)	3.029	1.929
Median (hours)	2.529	1.932
95% CI & CrI (hours)	[1.466, 7.683]	[1.741, 2.137]
Chloroquine (n)	174	176
Mean (hours)	3.599	2.861
Median (hours)	3.265	2.865
95% CI & CrI (hours)	[1.867, 7.324]	[2.689, 3.044]
*Severe malaria*		
Artemether–lumefantrine followed by artesunate (intravenous) (n)	15	16
Mean (hours)	2.485	1.970
Median (hours)	2.118	1.961
95% CI & CrI (hours)	[1.513, 4.867]	[1.622, 2.429]
Artesunate (intravenous) followed by artemether–lumefantrine (n)	77	87
Mean (hours)	2.532	1.792
Median (hours)	2.275	1.793
95% CI & CrI (hours)	[1.252, 4.181]	[1.584, 2.009]

^a^Worldwide Antimalarial Resistance Network Parasite Clearance Estimator

CI: Confidence interval; CrI: Credible interval

## Discussion

The quantification of parasite clearance is widely used to determine anti-malarial drug efficacy and to monitor the emergence of drug resistance. This study aimed to ascertain the most useful approach, considering the complexity and resources required, to determine the parasite clearance rate or half-lives of anti-malarials used to treat *P. knowlesi* malaria, using currently available tools. The data used were obtained from three clinical trials conducted in Sabah, Malaysia involving a total of 714 children and adults with knowlesi malaria.

Given the lack of drug pressure and high sensitivity to anti-malarial drugs, patients in *P. knowlesi* efficacy studies typically have a low number of parasitaemia measurements above the detection level using standard sampling schemes, suggesting Bayesian hierarchical modelling as an attractive alternative to the widely used WWARN PCE method. However, in this study, only 5% of patients were excluded in stage 1 of the WWARN PCE method. As expected, these 36 patients had three or fewer parasite measurements and a lower parasitaemia before treatment.

The data used in this analysis were derived from patients with uncomplicated and severe *P. knowlesi* malaria. The oral monotherapies artesunate–mefloquine and artemether–lumefantrine that were used to treat uncomplicated malaria had similar shorter median parasite clearance half-life estimates using method 1 (~ 2.6 h) while the anti-malarial drug chloroquine had a longer half-life of 3.2 h. Patients suspected or confirmed to have severe knowlesi malaria received a combination of intravenous artesunate and oral artemether–lumefantrine, and as expected, had a much higher initial parasite count per microlitre of blood compared to patients with uncomplicated malaria. However, these patients had a shorter median parasite clearance half-life estimate compared to patients with uncomplicated knowlesi malaria, who only received oral therapies, at 2.3 h versus 2.7 h. This finding is not surprising given *P. knowlesi* is highly sensitive to artesunate and with intravenous administration, there is no delay in absorption as may occur with the administration of oral medication.

There are advantages and disadvantages in using WWARN PCE (Method 1) and Bayesian hierarchical modelling (Method 2) to obtain estimates of parasite clearance rate (Table 4). While both methods have benefits, there are some drawbacks that need careful consideration prior to implementation.

**Table 4:** A list of pros and cons for Method 1 and Method 2

Characteristics	WWARN PCE (Method 1)	Bayesian ‘bhrcr’ package (Method 2)
Advantages	Simple and straightforward method that is user friendly Computationally fast	All patient data (including patients with ≤ 3 blood samples) are included in the analysis Has a robust sampling method that uses a changepoint model to determine the lag and tail phases accurately
Disadvantages	Requires frequent sampling for best results (e.g., recommends blood samples taken every 6-h) Omits patients from the second stage (calculation of population mean) if the parasite clearance rate cannot be calculated in stage 1 (due to fewer samples), potentially leading to selection bias Requires at least three consecutive and distinct positive blood sample measures above the microscopic detection limit per patient to successfully estimate parasite clearance rates	Complex method of calculation which requires in-depth statistical knowledge for execution, including understanding the hierarchical structure and prior distributions for each parameter included in the model Computational time for sampling from posterior distributions (e.g., about 10–36 h, depending on the number of samples and processing system used) Integral convergence issues may occur if there is too much variation between patients’ parasite clearance profiles Patients with frequent parasitaemia measurements contribute more information to the model parameter estimation leading to potential bias

Given the advantages and disadvantages, it is essential to determine which method is more suited to the targeted end user. For drug efficacy studies, the end user is most often researchers without the expertise on Bayesian hierarchical modelling to implement Method 2. As such, it is recommended that the standard two-stage approach (Method 1) be used to estimate parasite clearance half-lives or rates, especially when most of the patients have three or more positive and distinct blood sample measures. The standard two-stage method is computationally simple, reliable, and quick. While there were patient omissions when Method 1 was used in the study, it was a small proportion of the study population (36/714 (5%)). It is recommended that a table detailing the patient characteristics, the number of parasite count measurements per patient available and initial parasitaemia for those patient parasite profiles successfully analysed and for those excluded in stage 1 of Method 1 should be provided by researchers or clinicians to ensure the transparency of any selection bias.

Both methods for parasite clearance quantification were compared in an analysis using pooled data from three clinical trials conducted for knowlesi malaria. As such, a direct comparison of the degree of bias from each of the two methods could not be assessed, as this would require a simulation study. However, Fogarty et al. [29] have performed a simulation study comparing the two methods for *P. falciparum* malaria and concluded that the Bayesian estimator generally performed better, specifically in instances where patient profiles had lags and/or tail phases. Knowlesi malaria is not known to have drug selection pressure given its zoonotic nature, which means drug resistance is not a current concern here. While it could be worthwhile to identify the factors that contribute to the existence of lag and tail phases in *P. knowlesi* infections, in this study, there was only a very small percentage (3%) of patients with tail phases.

Lastly, there has been some debate as to whether parasite clearance half-lives or rates are the best measures for evaluating drug efficacy, given that dead parasites (i.e. killed by the anti-malarial drug) remain in blood circulation for some time before removal by the spleen, leading to an overestimation of live parasites counted via microscopy [40]. Instead, Khoury et al. suggest treating parasite removal and anti-malarial drug activity as two distinct activities to obtain more accurate results [40].

## Conclusions

Continual determination of anti-malarial drug efficacy is important to ensure optimal treatment regimens are being recommended and to monitor the emergence of drug resistance. One of the widely accepted ways to determine treatment efficacy is the use of parasite clearance curves that show the initial decrease in parasitaemia post-drug administration.

Based on this analysis of parasitaemia data from 714 patients enrolled in three separate clinical trials, it is recommended that the standard two-stage method (WWARN PCE) be used to quantify parasite clearance for patients infected with *P. knowlesi*. This recommendation is based on the user-friendliness of the software to implement this method. However, in the instance that most patient profiles have fewer than three blood samples with parasitaemia above the detection limit, the Bayesian hierarchical modelling method for analysis should be considered to obtain useful information about the parasite clearance from the scarce data available.

## Supplementary Information

**Additional file1: Figure S1.** Example of the traceplot before and after thinning for the population mean parasite clearance rate, $\gamma$. **Figure S2.** Example of the traceplot before and after thinning for the probability of lag and tail phases, ${\pi }^{\ell}$ and ${\pi }^{\tau }$. **Table S1. **Summary statistics for parasite clearance rates (/hour) using WWARN PCE and Bayesian hierarchical modelling methods. **Table S2.** Estimated parasite clearance rates by antimalarial treatment administered using WWARN PCE and Bayesian hierarchical modelling.

## Data Availability

The datasets used and/or analysed during the current study are available from the corresponding author on reasonable request.

## Abbreviations

WWARN: Worldwide Antimalarial Resistance Network
PCE: Parasite Clearance Estimator
ASMQ: Artesunate–Mefloquine
CQ: Chloroquine
AL: Artemether–Lumefantrine
AS: Artesunate
IQR: Interquartile range
CI: Confidence interval
CrI: Credible interval
BHM: Bayesian hierarchical models
μL: Microlitre
MCMC: Markov chain Monte Carlo
PCR: Polymerase chain reaction
WHO: World Health Organization
ACT: Artemisinin-based combination therapy
kg: Kilograms
IV: Intravenous
