# Supplementary material for: Quantification of parasite clearance in Plasmodium knowlesi infections
Source: Malar J. 2023 Feb 14;22:54. doi: 10.1186/s12936-023-04483-9 (PMC9926767; doi:10.1186/s12936-023-04483-9)
Supplement: Supplementary file 1 — Additional file1: Figure S1. Example of the traceplot before and after thinning for the population mean parasite clearance rate, $\gamma$γ. Figure S2. Example of the traceplot before and after thinning for the probability of lag and tail phases, ${\pi }^{\ell}$πℓ and ${\pi }^{\tau }$πτ. Table S1. Summary statistics for parasite clearance rates (/hour) using WWARN PCE and Bayesian hierarchical modelling methods. Table S2. Estimated parasite clearance rates by antimalarial treatment administered using WWARN PCE and Bayesian hierarchical modelling. [file 12936_2023_4483_MOESM1_ESM.docx]

Additional file

**Figure S1:** Example of the traceplot before (left) and after thinning (right, selecting every 50^th^ sample) for the population mean parasite clearance rate, $\gamma$.

**Figure S2:** Example of the traceplot before (left) and after thinning (right, selecting every 50^th^ sample) for the probability of lag and tail phases, $\pi^{\mathcal{l}}$ (a) and $\pi^{\tau}$ (b).

(a)

(b)

**Table S1:** Summary statistics for parasite clearance rates (/hour) using WWARN PCE^a^ and Bayesian hierarchical modelling methods.

| **Method** | **No. of patients** | **Mean (/hour)** | **Median (/hour)** | **IQR^b^ (/hour)** | **Range (/hour)** | **95% CI^c^ & CrI^d^ (/hour)** |  |
| --- | --- | --- | --- | --- | --- | --- | --- |
| **1** | 678 | 0.2635 | 0.2576 | 0.2012 - 0.3168 | 0.0472 - 0.8044 | [0.1093, 0.4596] |  |
| **2** | 714 | 0.3606 | 0.3388 | 0.2856 - 0.4506 | 0.0829 - 1.1824 | [0.1759, 0.6524] |  |

^a^ Worldwide Antimalarial Resistance Network’s Parasite Clearance Estimator

^b^ IQR - Inter-quartile range

^c^ CI - Confidence interval

^d^ CrI - Credible interval

**Table S2:** Estimated parasite clearance rates by antimalarial treatment administered using WWARN PCE^a^ and Bayesian hierarchical modelling.

| **Treatment** |  | **WWARN PCE^a^** | **Bayesian hierarchical modelling** |  |
| --- | --- | --- | --- | --- |
| **Uncomplicated malaria** | | | | |
| **Artemether-lumefantrine (n)** |  | **301** | **320** | |
| Mean (/hour) |  | 0.2718 | 0.3573 | |
| Median (/hour) |  | 0.2631 | 0.3575 | |
| 95% CI^b^ & CrI^c^ (/hour) |  | [0.1319, 0.4420] | [0.3276, 0.3834] | |
| **Artesunate-mefloquine (n)** |  | **111** | **115** | |
| Mean (/hour) |  | 0.2755 | 0.3594 | |
| Median (/hour) |  | 0.2740 | 0.3587 | |
| 95% CI & CrI (/hour) |  | [0.0905, 0.4727] | [0.3243, 0.3982] | |
| **Chloroquine (n)** |  | **174** | **176** | |
| Mean (/hour) |  | 0.2179 | 0.2422 | |
| Median (/hour) |  | 0.2123 | 0.2419 | |
| 95% CI & CrI (/hour) |  | [0.0947, 0.3714] | [0.2278, 0.2578] | |
| **Severe malaria** | | | | |
| **Artemether-lumefantrine followed by artesunate (intravenous) (n)** |  | **15** | **16** | |
| Mean (/hour) |  | 0.3148 | 0.3519 | |
| Median (/hour) |  | 0.3273 | 0.3535 | |
| 95% CI & CrI (/hour) |  | [0.1486, 0.4581] | [0.2854, 0.4273] | |
| **Artesunate (intravenous) followed by artemether-lumefantrine (n)** |  | **77** | **87** | |
| Mean (/hour) |  | 0.3069 | 0.3868 | |
| Median (/hour) |  | 0.3046 | 0.3866 | |
| 95% CI & CrI (/hour) |  | [0.1665, 0.5535] | [0.3450, 0.4376] | |

^a^ Worldwide Antimalarial Resistance Network Parasite Clearance Estimator

^b^ CI – Confidence interval

^c^ CrI - Credible interval
